# Giant hyperfine interaction between a dark exciton condensate and nuclei

**DOI:** 10.1126/sciadv.ado8763

**Published:** 2024-08-16

**Authors:** Amit Jash, Michael Stern, Subhradeep Misra, Vladimir Umansky, Israel Bar Joseph

**Affiliations:** ^1^ Department of Condensed Matter physics, Weizmann Institute of Science, Rehovot 7610001, Israel.; ^2^ Department of Physics, Bar-Ilan University, Ramat-Gan 5290002, Israel.

## Abstract

We study the interaction of a dark exciton Bose-Einstein condensate with the nuclei in gallium arsenide/aluminum gallium arsenide coupled quantum wells and find clear evidence for nuclear polarization buildup that accompanies the appearance of the condensate. We show that the nuclei are polarized throughout the mesa area, extending to regions that are far away from the photoexcitation area and persisting for seconds after the excitation is switched off. Photoluminescence measurements in the presence of radio frequency radiation reveal that the hyperfine interaction between the nuclear and electron spins is enhanced by two orders of magnitude. We suggest that this large enhancement manifests the collective nature of the *N*-exciton condensate, which amplifies the interaction by a factor of $\sqrt{N}$.

## Teaser

The electron-nucleus hyperfine interaction is shown to be strongly enhanced in an exciton condensate.

## INTRODUCTION

Excitons in semiconductors are expected to undergo a Bose-Einstein condensation (BEC) transition at low temperatures (*1*–*3*). Coupled quantum wells (CQWs) or bilayer structures, where electrons and holes are confined to distinct potential wells, have emerged as a leading platform for realizing this transition (*4*–*24*). The small overlap between the electron and hole wave functions, which bind to form spatially indirect exciton (IX), results in an extended exciton lifetime and a diminished spin relaxation rate (*25*) and allows reaching the critical density for condensation at relatively low power.

A substantial contribution to this endeavor, shedding light on the impact of the electron-hole exchange interaction, stems from the work of Combescot *et al.* (*9*, *12*). Their findings suggest that, because of this interaction, the optically inactive (spin-forbidden) dark exciton state is lower in energy relative to the optically active bright exciton and is therefore the ground state of the condensate. Consequently, above the condensation threshold, excitons are anticipated to predominantly occupy this long-lived ground state. Unfortunately, however, the lack of light emission from the condensate makes it difficult to study the properties of this dark condensate and confirm its collective nature. Experimental efforts have therefore focused on studying the behavior of the bright IXs in the presence of the condensate, particularly their blueshift above a certain threshold power and below a critical temperature (*10*). This blueshift, Δ*E*, which is due to the repulsive interaction between the IXs, was shown to manifest the buildup of a high exciton density in the dark ground state (*17*–*20*, *22*, *24*). It was shown that this repulsive interaction stabilizes the dark ground state by suppressing spin flipping IX-IX collisions up to very high densities (*22*, *24*). Other findings substantiating the occurrence of condensation take advantage of dark-bright exciton mixing, which turns the condensate to be partially radiative (*12*). These include observation of spatial coherence (*6*, *7*, *11*, *17*), spin polarization (*18*) and spin textures (*14*), defects proliferation representing quantized vortices (*21*), and enhanced luminescence (*23*).

**Fig. 1.**
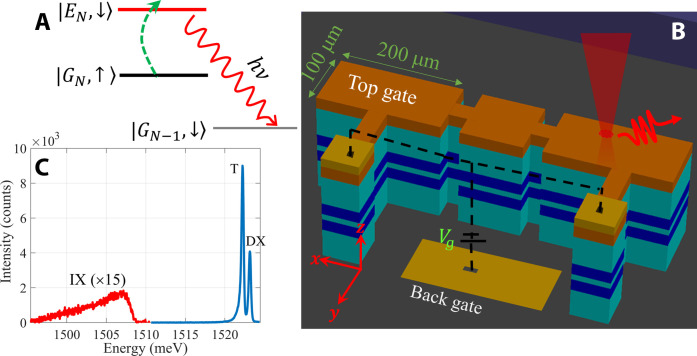
Device and spectra. (**A**) Schematic drawing of the radiatively assisted flip-flop transition. Here, *N* is the number of excitons. (**B**) Schematic drawing of the sample structure, where the blue strips in the center represent the CQW. (**C**) A typical PL spectrum at low power. DX, direct exciton; T, trion. Here, *V*_g_ = − 4 V, *P* = 0.5 μW, and *T* = 0.6 K.

In this work, we study the condensate hyperfine interaction with the nuclei and show that it can be an effective probe that reveals its collective properties. It is well known that the hyperfine interaction of optically excited electrons with nuclei in semiconductors may transfer the electrons spin to the nuclei, in a process known as dynamical nuclear polarization (DNP) (*26*–*28*). Numerous studies in the past two decades have demonstrated that the nuclei in a quantum dot can be effectively polarized by continuous resonant excitation with circularly polarized light (*29*–*35*). Here, we show that the continuous introduction of dark excitons, having a well-defined spin polarization, to the condensate ground state creates favorable conditions for DNP. The spin exchange (flip-flop) between an electron in the condensate ground state, ∣*G_N_*⟩, and a nucleus turns a dark exciton into bright, while transferring the electron spin to the nucleus. The bright exciton in the excited state, ∣*E_N_*⟩, is quickly removed from the system by radiative recombination, reducing the total number of excitons from *N* to *N* − 1 (Fig. 1A). The net effect of this process is flipping the nuclear spin from ↑ to ↓; repeating this process by pumping more and more dark excitons, we may create macroscopic nuclear polarization.

We investigate the photoluminescence (PL) and radio frequency (RF) spectrum of optically excited GaAs/AlGaAs CQW at low temperatures and find clear evidence for DNP that accompanies the appearance of the dark condensate. We show that the nuclei are polarized throughout the area of the mesa, extending to regions that are a few hundred micrometers from the excitation beam and persisting for a few seconds after the optical excitation is switched off. We find that the RF excitation of the condensate is in the tens megahertz range, ~10^2^ larger than the single-exciton hyperfine splitting. We suggest that this large enhancement is proportional to $\sqrt{N}$ , where *N* is the number of excitons in the condensate, allowing us to determine the condensate coherence volume.

The CQW structure consists of an 18-nm wide well (WW) and a 12-nm narrow well (NW), separated by a 3-nm Al_0.3_GaAs barrier. Top and bottom doped layers, which are 1 μm above and below the CQW, allow the application of a gate voltage, *V*_g_. Unlike many experiments on exciton BEC, which use an electrostatic trap or local disorder to confine the excitons in a small area, the samples we study in this work are large area mesa, ~10^5^ μm^2^ (Fig. 1B). We show that this structure allows dark excitons to diffuse away from the excitation region to the entire area of the mesa and polarize the nuclei throughout.

By tuning the excitation laser energy below the NW energy gap, we generate electrons and holes solely in the WW. Some of the photoexcited electrons may tunnel to the NW due to the applied electric field and bind to the holes in the WW to form IX, while the remaining electrons in the WW bind to these holes and form direct excitons or positively charged trions. The low-power PL spectrum displays these three lines: a direct exciton line at 1.524 eV, a trion line 1 meV below it, and a broad IX line further below, at an energy that depends on *V*_g_ (Fig. 1C) (*36*).

## RESULTS

### Dark exciton condensation and nuclear polarization

**Fig. 2.**
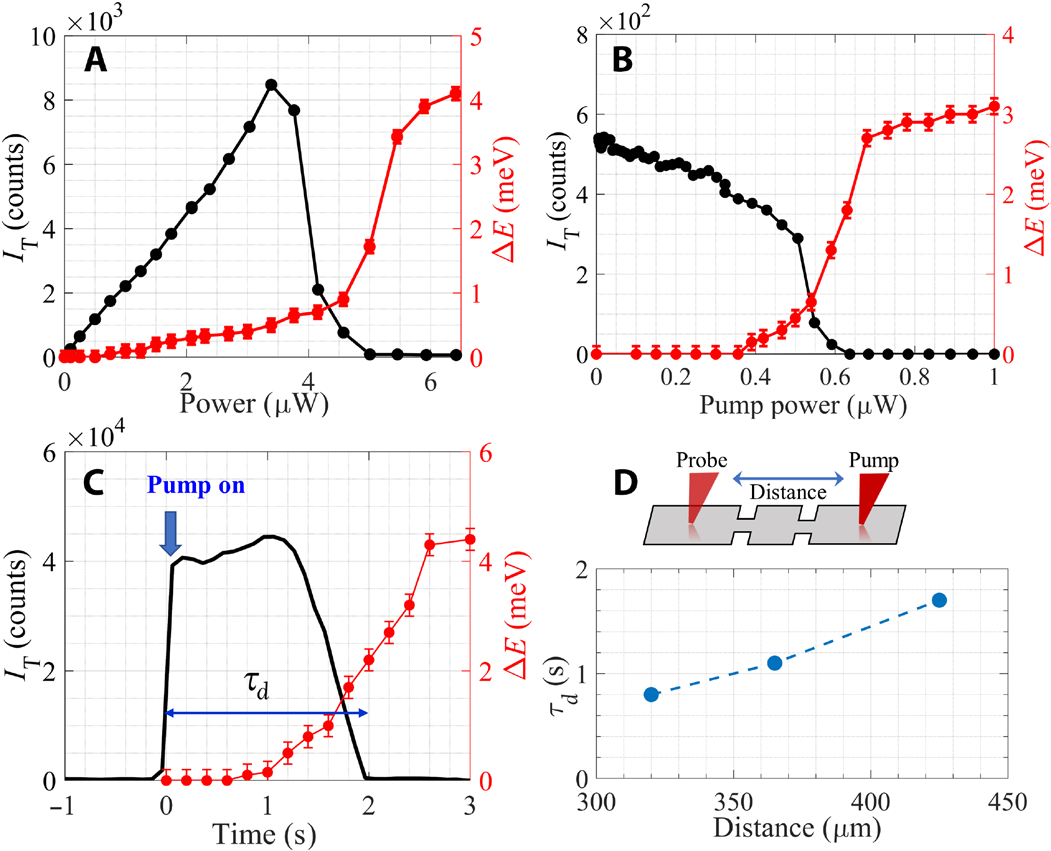
Dark exciton condensation and buildup of nuclear polarization. (**A**) The trion line intensity, *I*_T_, and the blueshift, Δ*E*, as a function of power at 1.5 K. The threshold is clearly visible. (**B**) *I*_T_ and ΔE at the probe location as function of pump power at *T* = 0.6 K (see schematic drawing). The threshold power is lower than in (A) due to the lower temperature. (**C**) The evolution of *I*_T_ and ΔE with time at 1.5 K. The delay time, τ_d_, is calculated from the onset of pump, *t* = 0, to when *I*_T_ = 0. (**D**) The delay time at different probe positions, where the excitation beam is fixed (origin) at 0.6 K.

We begin by presenting the evolution of the spectrum with power, showing that it exhibits a clear threshold behavior. Figure 2A shows a typical power dependence of the trion line intensity, *I*_T_, and IX energy blueshift, Δ*E*. It is seen that *I*_T_ first grows linearly with power and then sharply falls to zero at *P* = 5 μW. The IX exhibits corresponding changes, steeply blueshifting when *I*_T_ falls to zero (red curve in Fig. 2A). Since Δ*E* is proportional to the IX density, *n*_IX_, the threshold power, *P*_th_, at which it steeply rises signifies the point where *n*_IX_ sharply increases. The trion and IX compete on the same holes in the WW, and, therefore, the sharp increase in *n*_IX_ necessarily implies a drop of *I*_T_. Hence, we can use both the fall of *I*_T_ and the increase in Δ*E* to determine *P*_th_ (see section S4). We observe this behavior, namely, an abrupt fall of *I*_T_ and steep rise of Δ*E* above a threshold power, over a broad range of gate voltages and temperatures (*37*).

We can unambiguously show that the steep rise of Δ*E* above *P*_th_ is due to dark excitons density buildup. Figure 2B depicts the results of pump-probe measurement, where we excite the mesa at one end and measure the PL spectrum by a weak probe a few hundreds of micrometers away from the excitation spot. The spectrum at the probe location exhibits a strong dependence on pump power: Δ*E* sharply increases, and *I*_T_ falls when the pump power exceeds *P*_th_, implying that the IXs created by the pump have a very long lifetime (about hundreds of microseconds) and can therefore diffuse over long distances (*24*).

Figure 2C shows the time evolution of Δ*E* and *I*_T_ at the pump location following an abrupt turn-on of the excitation beam at a power level exceeding *P*_th_. It is seen that the increase in Δ*E* and corresponding decrease in *I*_T_ start after a long delay time, τ_d_, of ~1 s and reach their steady-state value after a few more seconds. We find that τ_d_ depends on the excess pump power above threshold, τ_d_ ∝ 1/(*P* − *P*_th_), such that at *P* ≈ *P*_th_, it may reach tens of seconds (see section S4). This slow dynamic is found over a broad parameter range of gate voltages and in various mesas of different shapes and dimensions from the same wafer, indicating that it manifests an intrinsic process (*16*).

These long response times in semiconductors are indicative of interaction with nuclear spins, which are known to relax at a very low rate through dipolar interaction with neighboring nuclei. Spin exchange that turns a dark exciton into bright is a loss mechanism for the condensate, which limits the occupation of the dark exciton ground state above threshold. As more and more nuclei become polarized with increasing time or pump power above threshold, this process is gradually quenched, and the dark exciton density may grow. Consequently, the rise time of the dark exciton density reflects the buildup time for the nuclear polarization to reach its steady state.

The open geometry, which allows dark excitons to diffuse away from the excitation region, implies that this nuclear polarization process occurs throughout the mesa. This is evident in the slow evolution of the probe spectrum in a pump-probe measurement. We find that the changes in *I*_T_ and Δ*E* at the probe location appear only after a long delay time, which increases as the distance between the pump and probe beams grows (Fig. 2D). In section S2, we present a simple one-dimensional diffusion model, which accounts for the DNP generated by the dark excitons and reproduces the observed slow dynamics.

### The giant hyperfine interaction between the condensate and nuclei

**Fig. 3.**
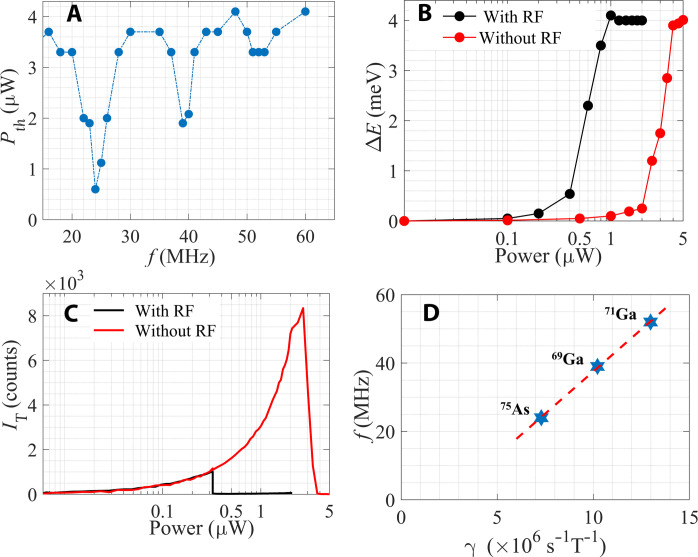
The RF resonances. (**A**) The threshold power, *P*_th_, as a function of RF. The three resonances at *f_i_*= 24, 39, and 52 MHz are clearly seen. (**B** and **C**) The blueshift, Δ*E*, and trion intensity, *I*_T_, as a function laser power without (red) and with (black) RF radiation at 24 MHz. (**D**) The dependence of resonance frequencies, *f_i_*, on gyromagnetic ratio γ*_i_* of the three isotopes in GaAs, ^75^As, ^69^Ga, and ^71^Ga. It is seen that *f_i_* values are exactly proportional to γ*_i_*.

To obtain further insight into the role of nuclear polarization in determining the optical spectrum, we performed power-dependent PL measurements while exposing the sample to RF radiation. The underlying idea is that at certain frequencies in the RF range, this radiation can be resonantly absorbed by the nuclei, causing them to flip their spin and thereby affect *P*_th_ (see section S1 for experimental details). In Fig. 3A, we show *P*_th_ as a function of RF, where each point is obtained by fixing the RF at a specific value and measuring the power dependence of the PL spectrum. We find pronounced dips in *P*_th_ at three resonance frequencies, *f_i_* = 24,39, and 52 MHz.

The impact of the RF radiation on the optical spectrum is explicitly demonstrated in Fig. 3 (B and C), which shows Δ*E* and *I*_T_ as a function of pump power with (black) and without (red) RF radiation at *f*_1_ = 24 MHz. We find that the resonance relative depth, defined as *P*_th_(*f*_1_)/*P*_th_(0), is proportional to the RF power and may reach two orders of magnitude reduction of *P*_th_ at the high RF power limit (see section S4).

The existence of three RF resonances at zero magnetic field suggests that they are associated with transitions between hyperfine levels of the three isotopes in the GaAs system: ^75^As, ^69^Ga, and ^71^Ga. The natural abundance of these isotopes, 100, 60, and 40%, respectively (*27*, *38*), matches the relative strength of the resonances. In Fig. 3D, we present the measured frequencies, *f_i_*, as a function of the gyromagnetic ratio of the three isotopes ( $\gamma_{\text{As}}^{75}=7.29$ , $\gamma_{\text{Ga}}^{69}=10.22$, and $\gamma_{\text{Ga}}^{71}=12.98\times 10^{6}$ s^−1^ T^−1^ (*38*)). It is seen that there is a perfect linear relationship between the two, indicating that *f_i_* are proportional to γ*_i_*. Unexpectedly, however, the values of *f_i_* are very high, ~10^7^ Hz, about two orders of magnitude larger than the ~10^5^-Hz hyperfine splitting expected for a GaAs exciton (*27*). We suggest below that this large enhancement of the electron-nucleus coupling is a manifestation of the collective interaction of the condensate and the nuclei.

Let us consider the collective hyperfine Hamiltonian, *H*_hf_, of *N* electrons in the exciton BEC ground state, each having +*s_z_* spin and coupled to a nucleus with a hyperfine coupling constant *g*. We can view the electrons as a collection of *N* two-level systems, each having an energy gap of *gs_z_I_z_* that depends on the nuclear spin, *I_z_*. *H*_hf_ can be expressed in terms of the total electron spin operator $S=\sum_{j=1}^{N}s^{j}$ , as *H*_hf_ = *g*[*S_z_ I_z_* + *S*_+_*I*_−_ + *S*_−_*I*_+_]. Since the system is in its BEC ground state, its collective wave function can be written as ∣*G_N_*, ↑⟩ = ∣ 0,0,0,0, …0⟩, and its collective excited state can be written as a superposition of single spin excitations, $|E_{N},↓\rangle =1/\sqrt{N}(\;|1,0,0,0\ldots ,0\rangle +|0,1,0,0\ldots ,0\rangle +|0,0,1,0\ldots ,0\rangle +\ldots )$. Here, the arrows ↑and↓mark the nuclear spin orientation at the initial and final states, respectively. It is easy to see that the matrix element between the two states is $\langle E_{N},↓\left|H_{\text{hf}}\right|G_{N},↑\rangle =\frac{1}{\sqrt{N}}\sum_{j=1}^{N}g/2=\sqrt{N}g/2$, and the energy difference between the collective ground and excited states is $\sqrt{N}g$ , enhanced by a large factor of $\sqrt{N}$ relative to the single particle case (see section S3 for a detailed derivation). Accordingly, the ~10^2^ enhancement factor implies that the number of excitons in the condensate is *N* ≈ 10^4^ to 10^5^. Taking the density to be ~10^10^ cm^−2^, the coherence length can be estimated to be ~10 μm. This coherence length scale is consistent with the fact that the condensate extends to a few hundreds of micrometers (Fig. 2D).

### The hyperfine interaction in a magnetic field

Support for this interpretation is provided by measurements in a magnetic field, *B*_ext_, oriented along the +*z* direction. The field breaks the degeneracy between the ∣ ± 2⟩ dark exciton states, such that the electron polarization in the condensate ground state is $|+\frac{1}{2}\rangle \;$ , and the energy gap to the excited state at threshold is the Zeeman energy, *ℏ*ω_e_. However, spin flip transitions from dark to bright excitons polarize the nuclei along the +*z* direction, yielding an Overhauser field, *B*_O_, on the electrons that is oriented opposite to *B*_ext_ and closing the Zeeman gap (*30*). As the pump power is increased, more and more nuclei are polarized, and *B*_O_ grows until it compensates the external field, *B*_O_ = − *B*_ext_ (*39*, *40*). Increasing the pump power beyond this value will not result in further polarization of the nuclei, and the dark exciton density may start growing.

**Fig. 4.**
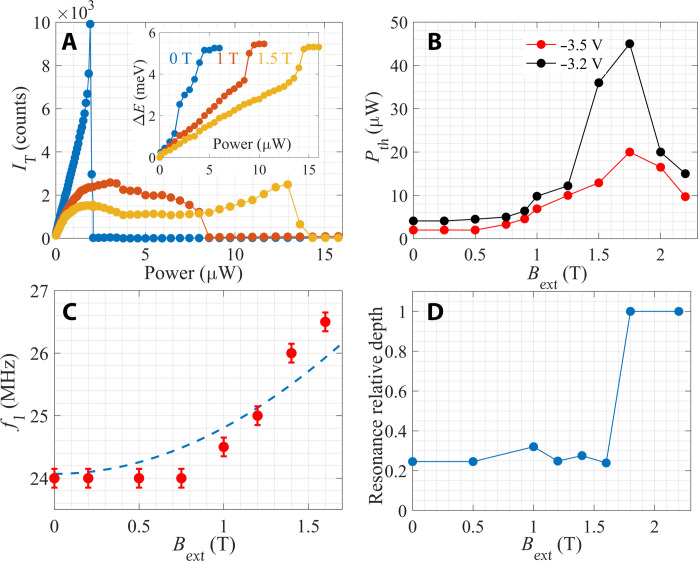
The behavior in a magnetic field *B*_ext_. (**A**) Trion intensity, *I*_T_, and blueshift, Δ*E*, as function of laser power at *B*_ext_= 0 (blue), 1 T (orange), and 1.5 T (yellow). *V*_g_ = − 3.5 V and *T* = 1.5 K. The intermediate range, at which *I*_T_ is approximately constant, is clearly seen. (**B**) *P*_th_ as a function of magnetic field for two different gate voltages at *T* = 1.5 K. (**C**) The ^75^As resonance, *f*_1_, as a function of magnetic field. The dashed line shows the predicted behavior. (**D**) The resonance relative depth, *P*_th_(*f*_1_)/*P*_th_(0), as a function of magnetic field at *T* = 1.5 K. The resonance disappears at *B*_ext_ > 1.75 T.

The evolution of the spectrum in a magnetic field nicely shows this behavior. Figure 4A depicts *I*_T_ and Δ*E* as a function of power for several magnetic fields. It is seen that the saturation power of *I*_T_, which marks the condensation onset at *P* ≈ 2 μW, is almost independent of magnetic field. However, it is evident that there is an intermediate broad power range following this onset, in which *I*_T_ remains approximately constant. Only beyond this power range, *I*_T_ falls to zero, and Δ*E* exhibits a steep rise. This intermediate power range is the range at which the excess power goes to polarizing the nuclei. Accordingly, the power at which *I*_T_ = 0 and Δ*E* exhibits a steep rise is the point at which *B*_O_ = − *B*_ext_.

The increase in *P*_th_ in the range 0 < *B*_ext_ < 1.75 T seen in Fig. 4 (A and B) agrees with this predicted behavior and reflects the growing number of polarized nuclei that are needed to compensate the field with increasing *B*_ext_. The sharp drop of *P*_th_ at *B*_ext_ > 1.75 T is particularly significant. We note that when all nuclear spins in GaAs are polarized, the Overhauser field is $B_{O}^{\text{max}}=5.3$ T (*27*, *28*, *38*). Therefore, the peak in *P*_th_ at $B_{\text{ext}}\approx -B_{O}^{\text{max}}/3$ corresponds to the case when all spins projections, which were initially aligned randomly along the ±*z*, are oriented along +*z*. This is the maximal nuclear polarization that can be induced by electrons. Beyond this point, the Overhauser field cannot grow further, and the dark to bright conversion is suppressed.

Let us now examine the effect of RF radiation when the system is subjected to an external magnetic field. Diagonalizing *H*_hf_ in this case, the excitation energy is given by $\sqrt{Ng^{2}+{\left(\omega_{e}-\omega_{n}\right)}^{2}}$ , where ω_e_ and ω_n_ are the electron and nuclear Zeeman splitting, respectively (see section S3). We first consider the *B*_ext_ < 1.75 T case, where *B*_O_ = −*B*_ext_, and we can therefore set ω_e_ = 0. In this range the resonance frequency should be given by $f_{i}\left(B\right)=\sqrt{f_{i}^{2}+{\left(\gamma_{i}B_{\text{ext}}\right)}^{2}}$ . In Fig. 4C, we compare the measured dependence of the *f*_1_ resonance on *B*_ext_ with the predicted behavior taking the value for ${\text{γ}}_{\text{As}}^{75}$ . It is seen that there is a good qualitative and quantitative agreement.

When *B*_ext_ > 1.75 T, the external field cannot be canceled by *B*_O_, and there is a net magnetic field acting on the electron. Since ω_e_ is much larger than both ω_n_ and $\sqrt{N}g$ , the excitation energy of the condensate can be approximated by ≈*ℏ*ω_e_, which is in the gigahertz frequency range. In this case, we do not detect any measurable effect of the RF radiation on *P*_th_ in the range of 0 to 100 MHz. This is demonstrated in Fig. 4D, which shows the relative depth of the first resonance as a function of *B*_ext_. The abrupt disappearance of the resonance at 1.75 T is clearly visible.

## DISCUSSION

The observation of high-frequency RF resonances, which are two orders of magnitude larger than the single-exciton hyperfine resonances, and their nontrivial dependence on magnetic field is well explained by a collective model of *N* electrons in the exciton BEC ground state, each coupled to a nucleus. In that sense, these resonances may be considered as evidence for the existence of an exciton condensate and an effective tool to study it despite its dark nature.

We note that the $\sqrt{N}$ enhancement of the hyperfine coupling is similar to that found in the Tavis-Cummings model, describing the energy spectrum of *N* spins interacting with a radiation field (*41*, *42*). The two important properties of that model are present also here: The interaction Hamiltonian commutes with the total electron spin operator, and the system of *N* spins is in its ground state. In that sense, the occurrence of these resonances confirms the collective nature of the excitons and provides important insights. Note that the fact that the nuclei are polarized throughout the mesa and the persistence of this polarization for a long time after the optical excitation is switched off may offer an interesting system for quantum information applications using CQW or bilayer structures.

## MATERIALS AND METHODS

The CQW consist of two quantum wells having widths of 12 and 18 nm and a barrier of 3-nm Al_0.28_Ga_0.72_As. The CQW is embedded between two superlattices of ~1 μm in thickness each. The superlattice below the CQW has 33 periods of [27-nm Al_0.37_Ga_0.63_As + 2-nm AlAs + 1-nm GaAs], and that above the CQW has 20 periods of [50-nm Al_0.37_Ga_0.63_As + 1-nm GaAs]. The top (0.15 μm) and bottom (0.25 μm) layers are silicon-doped (*n* = 10^18^ cm^−3^) Al_0.12_Ga_0.88_As. Mesas of various shapes and sizes were prepared using optical lithography and wet etching after the molecular beam epitaxy growth of the sample. Most of the data presented in this work were collected using an elongated structure, which is depicted in Fig. 1.

The experiments were conducted using two different cryostats: a dilution refrigerator with optical access, which allows conducting PL measurements in the range of 100 mK to 5 K, and a split-coil magneto-optical pumped-helium cryostat, with a temperature range of 1.5 to 10 K and magnetic fields up to 7 T. Throughout the measurements, we explored a temperature range of 100 mK to 6 K and a magnetic field range of 0 to ±5 T.

For the single-beam measurements, we illuminated the sample using a Ti:sapphire laser with a Gaussian spot size of σ = 20 μm and an energy of 1.5287 eV. We have adjusted the laser energy to be below the energy gap of the NW such that carriers are created in the WW only. In the pump-probe experiment, a diode laser with the same Gaussian spot size and an energy of 1.5794 eV was used as the probe beam. To collect PL from different regions of the mesa, a pinhole with a diameter of 50 μm was positioned at the objective’s focal plane. The PL from the sample was guided to a Spex (500 M) spectrometer and imaged and analyzed using an Andor iXon camera. A Keithley 2400 source meter was used to apply the gate voltage. LabVIEW and Andor software are used for the acquisition and recording of all the experimental measurements. To generate the RF field across the sample, an ac current is applied to a coil that is fixed to a 24-pin chip header with epoxy. Further details about the RF measurements are discussed in the Supplementary Materials.

## Supplementary Materials

### This PDF file includes:

Sections S1 to S4 Figs. S1 to S9 References
