# Supplementary material for: Giant hyperfine interaction between a dark exciton condensate and nuclei
Source: Sci Adv. 2024 Aug 16;10(33):eado8763. doi: 10.1126/sciadv.ado8763 (PMC11328897; doi:10.1126/sciadv.ado8763)
Supplement: Supplementary file 1 — Sections S1 to S4 Figs. S1 to S9 References [file sciadv.ado8763_sm.pdf]

Science Advances
AAAS

Supplementary Materials for

Giant hyperfine interaction between a dark exciton condensate and nuclei

Amit Jash *et al.*

Corresponding author: Amit Jash, amit-kumar.jash@weizmann.ac.il

Sci. Adv. **10**, eado8763 (2024)
DOI: 10.1126/sciadv.ado8763

This PDF file includes:

Sections S1 to S4
Figs. S1 to S9
References

1. RF Measurements Setup

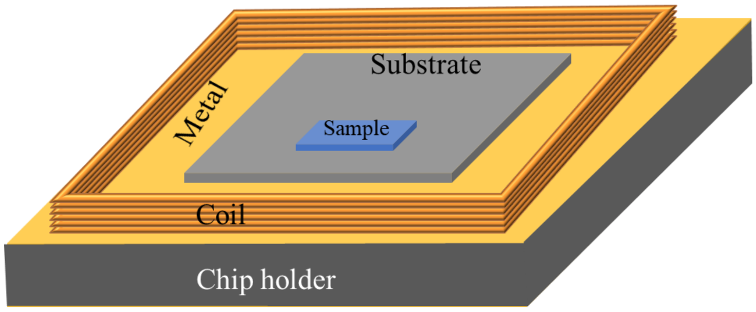

Fig. S1. A schematic drawing of the RF measurements setup.

The configuration, which was implemented to generate an oscillating magnetic for the RF measurements, is described in Fig. S1. The coil, which has six layers with five turns each, was wound on a bobbin prepared using a 3D printer, employing PVA material. Once the coil was wound, the PVA material was dissolved by water, and the coil was fixed to a 24-pin chip header with epoxy. We note that the metal surface of the chip holder strongly suppresses the z component of the RF field at the area near the center of the coil. Hence, by placing the mesa a little off-center, we obtain a component of the RF field in the CQW plane. Indeed, we verified that similar results were obtained when the coil was oriented in a perpendicular direction.

To generate RF magnetic field, we employed a function generator from Berkeley Nucleonics Corp (Model A2255), which has a frequency range of 1-250 MHz and an adjustable amplitude of 1-10 V. Throughout the RF measurement process, we took caution to prevent excessive heating of the sample. Indeed, we did not observe any such effects. The current flowing through the coil remained in the range of a few milliamperes, resulting in RF radiation at the sample surface within the nanowatt range. To determine P_{th} at a particular frequency, we fixed the RF radiation at a frequency within the $0 - 100$ MHz range and measured the PL spectrum as the power is ramped up at a slow rate, waiting 5 seconds between consecutive measurements. To obtain a curve like Fig. 3A we repeated this process in steps of 0.5 MHz over a broad RF range.

2. Diffusion Model

To model the dark condensate – nuclear interaction we conducted a simple one-dimensional diffusion simulation of the following couples equations for the dark exciton density, n, and polarized nuclear density, N

$$\frac{\partial n}{\partial t} = P - \frac{n}{\tau_{nr}} + D\frac{\partial^2 n}{\partial x^2} - \alpha n(N_0 - N) \tag{S1}$$

$$\frac{\partial N}{\partial t} = -\frac{N}{\tau_N} + \alpha n(N_0 - N) \tag{S2}$$

Here $P = P_0 \exp\left(-\frac{x^2}{\sigma^2}\right)$ is the excitation pump beam, which is assumed to have a gaussian profile, τ_{nr} is the non-radiative recombination time of dark excitons, D is the exciton diffusion constant, N_0 is the total nuclear density, τ_N is the nuclear relaxation time, and α is the coupling term, which polarizes the nuclei and causes dark exciton to disappear by turning it into bright.

In Fig. S2 we show the time evolution of the exciton density, n, and nuclear polarization, N, at the pump location. The delay time, τ_d, which characterized the buildup of in n and N is clearly seen.

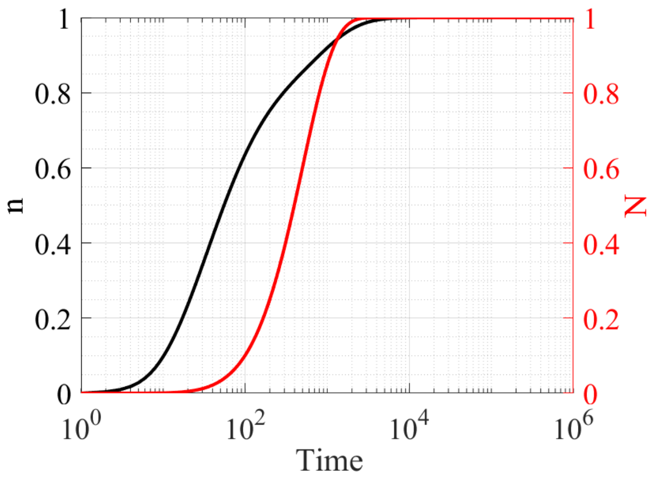

Fig. S2. The diffusion model. The evolution of exciton density and nuclear polarization with time.

3. Hyperfine Coupling of Nuclei with a Condensate

a. Hyperfine interaction between single nucleus and single electron

Let's first consider a single nucleus coupled to a single electronic spin by hyperfine interaction. The Hamiltonian of the system is given by,

$$H = \frac{1}{2}\hbar\omega_n\sigma_{z,n} + \frac{1}{2}\hbar\omega_e\sigma_{z,n} + \hbar g/4\boldsymbol{\sigma_e}.\boldsymbol{\sigma_n} \tag{S3}$$

Here σ_i are the Pauli matrices, g is the hyperfine constant, and $\omega_{e,n}$ are the cyclotron frequency of electron and nucleus, respectively, in a magnetic field *B*.

This Hamiltonian can be written as

$$H = \frac{1}{2}\hbar\omega_n(I \otimes \sigma_{z,n}) + \frac{1}{2}\hbar\omega_e(\sigma_{z,e} \otimes I) + \frac{1}{4}\hbar g(\sigma_{z,e} \otimes \sigma_{z,n}) + \frac{1}{2}\hbar g(\sigma_e^+\sigma_n^- + \sigma_e^-\sigma_n^+) \tag{S4}$$

I is unit matrix Denoting the electron and nuclear states by $\pm$ and $\uparrow, \downarrow$, respectively, the Hamiltonian can be represented in the basis $|+,\uparrow>, |+,\downarrow>, |-,\uparrow>$, and $|-,\downarrow>$ as

$$H = \frac{\hbar}{2}\begin{pmatrix} \omega_e + \omega_n + g/2 & 0 & 0 & 0 \\ 0 & \omega_e - \omega_n - g/2 & g & 0 \\ 0 & g & -\omega_e + \omega_n - g/2 & 0 \\ 0 & 0 & 0 & -\omega_e - \omega_n + g/2 \end{pmatrix}$$

The eigenvalues of this Hamiltonian are

$$E_{+\uparrow} = \frac{\hbar}{2}\left[\omega_e + \omega_n + \frac{g}{2}\right]$$

$$E_{+\downarrow} = \frac{\hbar}{2}\left[\sqrt{(\omega_e - \omega_n)^2 + g^2} - \frac{g}{2}\right]$$

$$E_{-\uparrow} = \frac{\hbar}{2}\left[-\sqrt{(\omega_e - \omega_n)^2 + g^2} - \frac{g}{2}\right]$$

$$E_{-\downarrow} = \frac{\hbar}{2}\left[-\omega_e - \omega_n + \frac{g}{2}\right]$$

The NMR transitions correspond to

$$\Delta E_1 = E_{+\uparrow} - E_{+\downarrow} \simeq \hbar(\omega_n + g/2)$$

$$\Delta E_2 = E_{-\uparrow} - E_{-\downarrow} \simeq \hbar(\omega_n - g/2)$$

The ESR transitions correspond to

$$\Delta E_3 = E_{+\uparrow} - E_{-\uparrow} \simeq \hbar(\omega_e + g/2)$$

$$\Delta E_3 = E_{+\downarrow} - E_{-\downarrow} \simeq \hbar(\omega_e - g/2)$$

where we took into account that $\omega_e - \omega_n >> g$.

b. Hyperfine interaction between a nuclear spin and with the electrons of an exciton BEC

Using Eq. (S4), one can write the Hamiltonian of the system

$$H = \frac{1}{2}\hbar\omega_n(I \otimes \sigma_{z,n}) + \frac{1}{2}\hbar\omega_e(\sum\sigma_{z,e}^{(i)} \otimes I) + \frac{1}{4}\hbar g(\sum\sigma_{z,e}^{(i)} \otimes \sigma_{z,n}) + \frac{1}{2}\hbar g\sum(\sigma_{e,(i)}^{+}\sigma_n^{-} + \sigma_{e,(i)}^{-}\sigma_n^{+}) \qquad \text{(S5)}$$

Using the total electron spin operator $S = \sum\sigma_e^{(i)}$ the Hamiltonian can be written as

$$H = \frac{1}{2}\hbar\omega_n\sigma_{z,n} + \frac{1}{2}\hbar\omega_e S_z + \frac{1}{4}\hbar g S_z \otimes \sigma_{z,n} + \frac{1}{2}\hbar g(S_+\sigma_{-n} + S_-\sigma_{+n}) \qquad \text{(S6)}$$

The electronic system is supposed to be initially in its ground state $|G\rangle = |-,-,-,\dots-\rangle$ of N electrons. The first excited state of the system consists of a single electron spin flip and can be written as $|E\rangle = 1/\sqrt{N}(|+,-,-,\dots-\rangle + |-,+,-,\dots-\rangle + \cdots|-,-,-,\dots+\rangle)$ *(43)*.

We can represent the Hamiltonian of the system in the subspace $|G,\uparrow>, |G,\downarrow>, |E,\uparrow>$, and $|E,\downarrow>$ as 4×4 matrix with out of the diagonal matrix element

$$\langle E,\downarrow|H|G,\uparrow\rangle = \frac{1}{\sqrt{N}}\frac{\sum\hbar g}{2} = \sqrt{N}\,\hbar g/2$$

And thus

$$H = \frac{\hbar}{2}\begin{pmatrix} \omega_e + \omega_n + g/2 & 0 & 0 & 0 \\ 0 & \omega_e - \omega_n - g/2 & g\sqrt{N} & 0 \\ 0 & g\sqrt{N} & -\omega_e + \omega_n - g/2 & 0 \\ 0 & 0 & 0 & -\omega_e - \omega_n + g/2 \end{pmatrix}$$

with eigen energies:

$$E_{E\uparrow} = \frac{\hbar}{2}\left[\omega_e + \omega_n + \frac{g}{2}\right]$$

$$E_{E\downarrow} = \frac{\hbar}{2}\left[\sqrt{(\omega_e - \omega_n)^2 + Ng^2} - \frac{g}{2}\right]$$

$$E_{G\uparrow} = \frac{\hbar}{2}\left[-\sqrt{(\omega_e - \omega_n)^2 + Ng^2} - \frac{g}{2}\right]$$

$$E_{G\downarrow} = \frac{\hbar}{2}\left[-\omega_e - \omega_n + \frac{g}{2}\right]$$

Note that we kept the labelling of the energy levels, even though the hyperfine interaction mixes the states $|G,\uparrow>$ and $|E,\downarrow>$. The energy difference between $E_{E\downarrow}$ and $E_{G\uparrow}$ is:

$$\Delta E = E_{E\downarrow} - E_{G\uparrow} = \hbar\left[\sqrt{(\omega_e - \omega_n)^2 + Ng^2}\right] \qquad \text{(S7)}$$

At zero magnetic field it becomes $\Delta E = \hbar\sqrt{N}g$.

We wish to comment here that the linear fit of Fig. 3D does not extrapolate to zero at $\gamma = 0$. This implies that the measured resonances are shifted by -12 MHz, independent of the isotope involved. We do not know the reason for this shift.

4. Extended Data

a. Gate voltage dependence

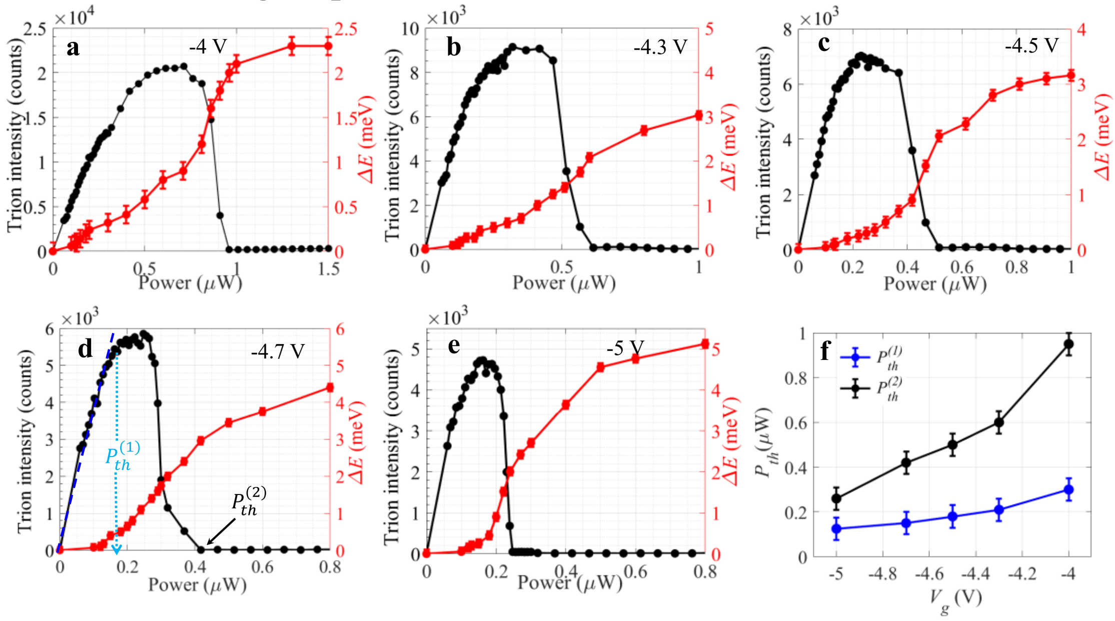

Fig. S3. Threshold power with gate voltage. (a-e) The trion intensity (left axis) and blueshift (right axis) as function power at 0.6 K at different gate voltage. In Fig. (d), the blue line depicts the linear relationship between trion intensity and power. The extraction of threshold power, $P_{th}^{(1)}$ and $P_{th}^{(2)}$, from the data, using two methods is presented in Fig. (d). (f) The measured P_{th} for the two methods as a function of gate voltage at 0.6 K.

Figure S3(a-e) illustrates the trion intensity, I_T, and blueshift, ΔE, as functions of laser power for various gate voltages at 0.6 K. The electrons from the wide well (WW) tunnel to the narrow well (NW) under the influence of gate voltage. The tunneling time closely compares with the direct exciton (DX) formation time, causing some photoexcited electrons to remain in the WW and form direct excitons. Excess holes in the WW have two options: they can either bind to the electrons in the NW, forming indirect excitons (IX), or bind to the excitons, forming trions (T). At very low power, below 100 nW, the favorable condition for holes is to bind to excitons and form trions rather than forming IX with NW electrons. At a certain threshold power, labeled as $P_{th}^{(1)}$ in Fig. S3(d), the trion intensity, I_T, deviates from linear increase with power and saturates. At this point, holes start binding with the NW electrons and the IX density increases. This is manifested as a blueshift of the IX energy, ΔE. Simultaneously, the trion intensity drops and eventually reaches zero. We label the power at which I_T becomes zero as $P_{th}^{(2)}$. In

Fig. S3(f) we show the gate voltage dependence of the two threshold powers. It is evident that the two curves differ by a constant scaling factor. Since the point at which $I_T = 0$ can be easily and unambiguously determined, we chose it as the definition of P_{th}.

The two methods of extracting threshold yield slightly different critical condensation density, n_c. Using $P_{th}^{(1)}$ we obtain $\Delta E = 0.4$ meV at 0.6K, which for uncorrelated excitons corresponds to $n_c \approx 1.6 \times 10^9$ cm^{-2}. This value is in good agreement with the theoretical value, $n_c = \frac{M}{h^2} k_B T$, which gives $n_c \approx 2 \times 10^9$ cm^{-2}.

b. Temperature dependence

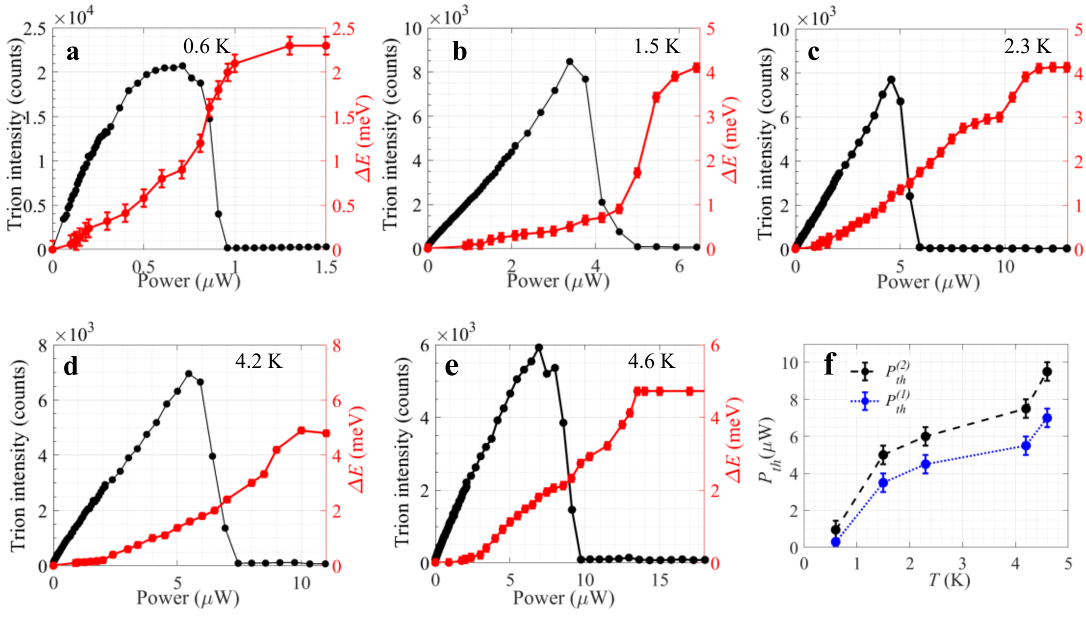

Fig. S4. Threshold power with temperature. (a-e) The trion intensity and blueshift as function of laser power at V_g = - 4 V for different temperature. (f) $P_{th}^{(1)}$ and $P_{th}^{(2)}$ as a function of temperature. (f) The measured P_{th} for the two methods at - 4 V.

Figure S4(a-e) displays the trion intensity and blueshift as a function of excitation power at four different temperatures: 0.6 K, 1.5 K, 2.3 K, 4.2 K, and 4.6 K. Note that the measurements at 0.6 K were performed with improved collection system, yielding a 10-fold increase in the PL intensity). It is clearly seen that P_{th} increases with temperature, and this is manifested both in $P_{th}^{(1)}$ and in $P_{th}^{(2)}$.

c. Condensation density

The critical density for condensation, n_c, is expected to increase linearly with temperature, $n_c = \frac{M}{h^2} k_B T$, where k_B and h are the Boltzmann and Planck constants, respectively, and M is the exciton mass. Indeed, we find that ΔE at threshold, which is proportional to n_c, increases linearly with temperature (Fig. S5(a)) and is nearly independent of gate voltage at constant temperature (Fig. S5(b)).

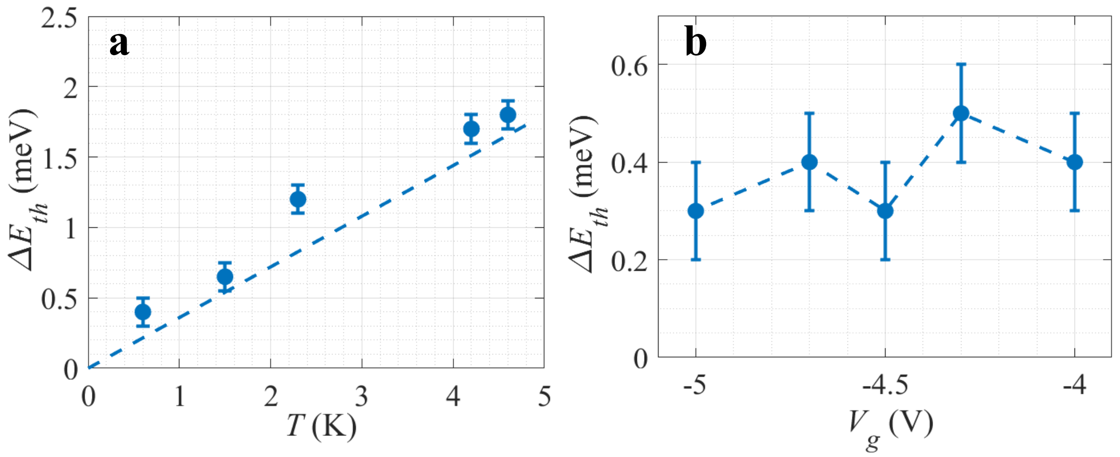

Fig. S5. The condensation density with temperature and gate voltage. The threshold blueshift, ΔE_{th}, as function of (a) temperature at $V_g = -4.0$ V and (b) gate voltage at 0.6 K. The points are taken where trion intensity deviates from linear behaviour ($P_{th}^{(1)}$).

d. Slow build-up of the dark excitons density

Figure S6(a-c) presents the time evolution of the spectrum for various laser powers at $T = 1.5$ K and $V_g = -4$ V, following switching on the excitation laser at $t = 0$. The two high energy lines are the direct exciton (DX) and trion (T). The weak IX line is outlined by white dashed line. It is seen that after a delay time (τ_d) of a few seconds, which decreases with increasing power, the trion line vanishes, and the IX energy undergoes an abrupt blueshift. It should be noted that at later times, the intensity of the DX line diminishes, and a new line labelled as Z emerges at a lower energy and eventually becomes the dominant line (*16*,*44*). In this study, we focus on the power range below the emergence of the Z line.

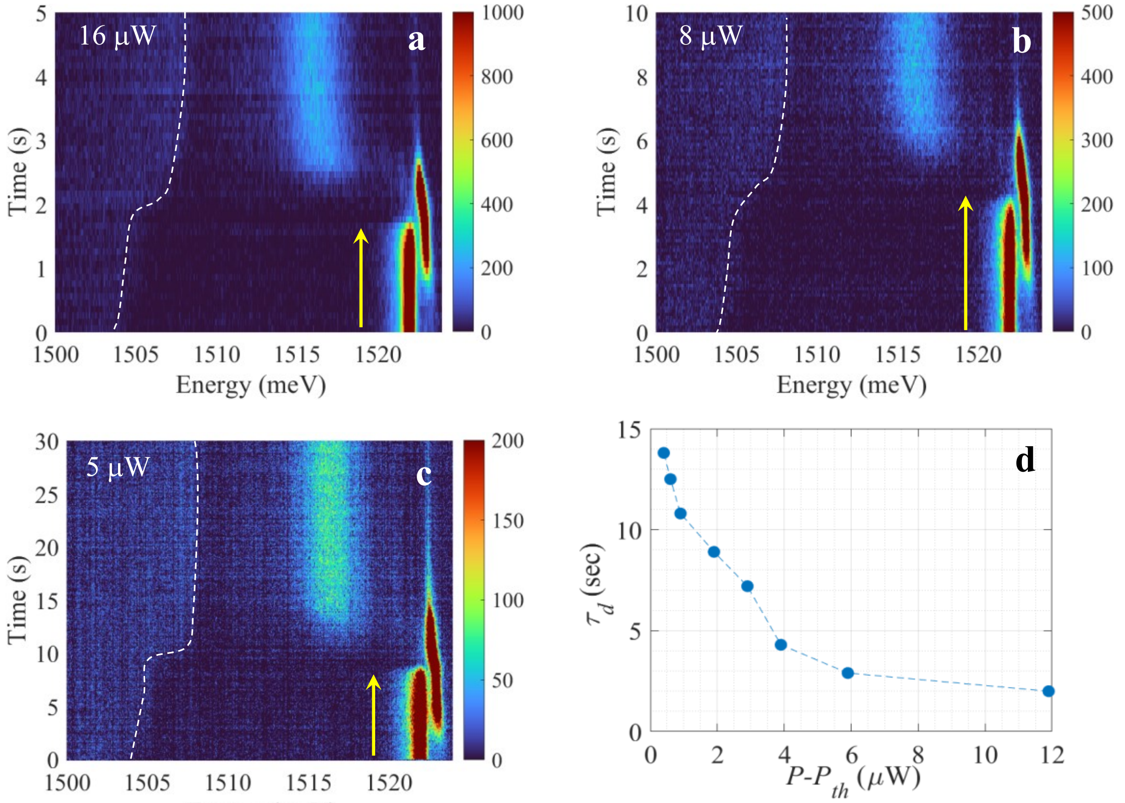

Fig. S6. Evolution of the spectrum with time. (a-c) The time evolution of the spectrum for various laser powers at T = 1.5 K and V_g = - 4 V. The IX energy is outlined by white dashed line. (d) The delay time (τ_d) as function of excess pump power.

e. The decay of nuclear polarization

To determine the decay of the nuclear polarization, we conduct pump-probe measurements, where we follow the recovery of the spectrum at the probe position following an abrupt switching off the pump power. The experiment was conducted using a 5 μW pump and 50 nW probe (P_{th}= 0.9 μW). We first turned on the pump for 50 seconds, letting the sample to reach its steady state, and then turned it off at $t = 0$ (as depicted in Fig. S7). We find that the trion line intensity (I_T) began to increase approximately 3 seconds after the pump was turned off and reached its saturated value after more than ten seconds. The slow recovery of the trion line marks the decay of nuclear polarization at the probe position.

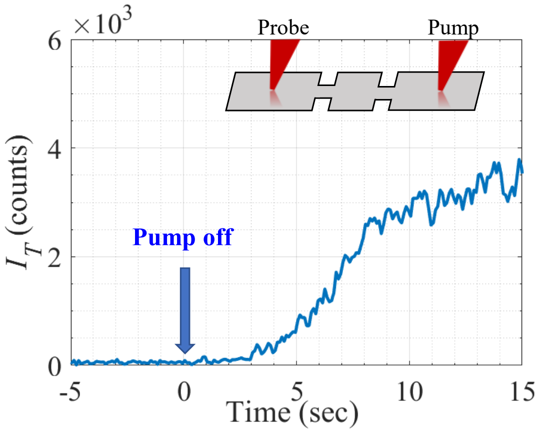

Fig. S7. The delay time at the probe position. The time evolution of the trion line intensity, I_T, at the probe location after the pump is switched off at $t = 0$. Here T = 0.6 K and V_g = -4 V.

f. RF measurements

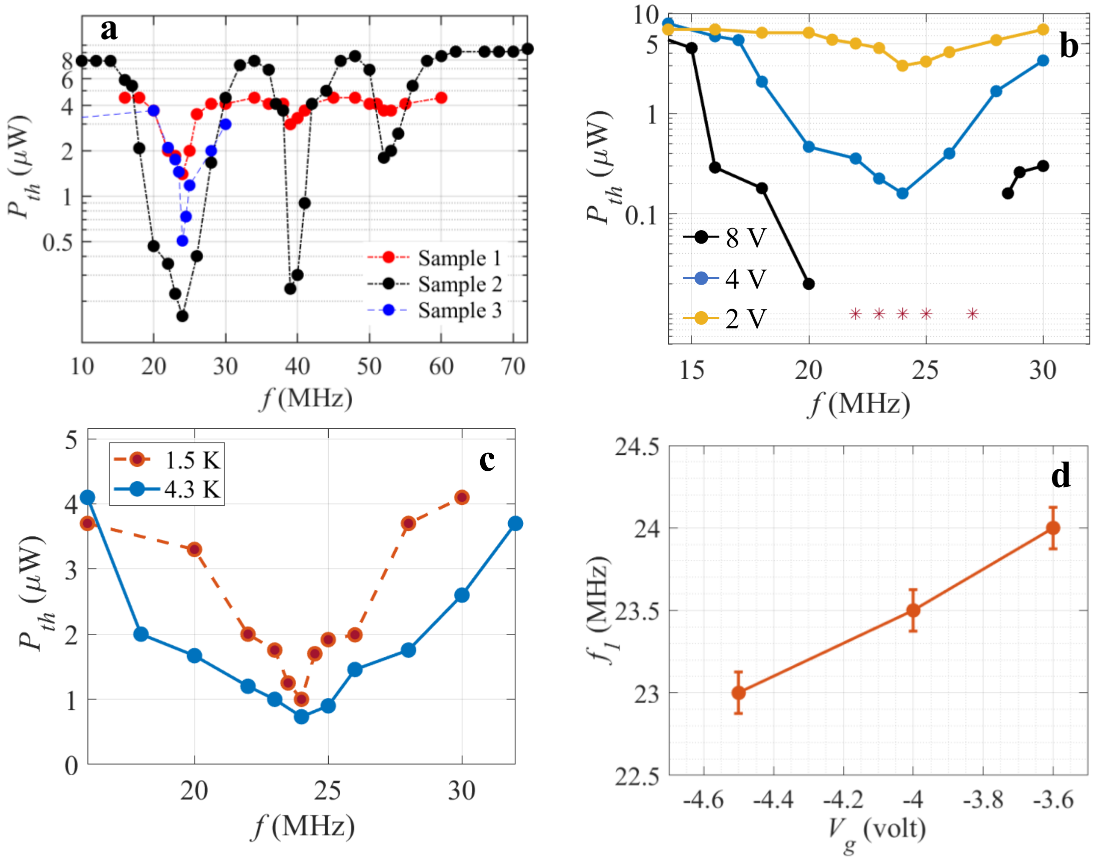

Fig. S8. Measured P_{th} under RF excitation. The measured P_{th} as function of RF frequency at T=1.5K and V_g= -4V; (a) at three different samples; (b) at three RF voltages in sample 2; (c) at two different temperatures with a fixed V_g of - 4 V. (d) The first resonance different with gate voltage at 1.5 K.

Figure S8 illustrates the variation the RF resonances as we change various parameters. In (a) we show the RF resonances for three different mesas. It is evident that while the resonance depth varies, the resonance frequency is the same. In (b) we show how P_{th} becomes lower at resonance with increasing RF power. At the highest RF voltage in the range $20 - 27$ MHz, P_{th} is lower than 10 nW and could not be determined accurately (shown by star symbol). In (c) we show the resonance in two temperatures, 1.5 and 4.3 K. We can see that the resonance is broadened at higher temperature, but the resonance frequency remains the same. Finally, in (d) we demonstrate the dependence of the resonance frequency on gate voltage. We notice a small shift of the resonance to lower frequency as $|V_g|$ increases. We believe that this reflects the change of the electron wavefunction, ψ, which is pushed into the barrier and thereby reducing the factor $|\psi(0)|^2$ in the hyperfine coupling.

g. Voigt and Faraday configurations

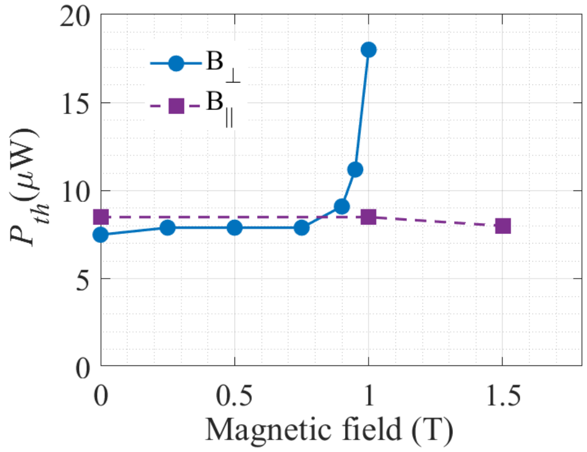

Fig. S9. P_{th} with magnetic field. P_{th} as function of magnetic field for Voigt, $B_\parallel$, and Faraday, $B_\perp$, configurations. Here T = 1.5 K and $V_g = -4$ V (measurements were performed on sample 2).

Figure S9 compares the measured P_{th} values in Voigt and Faraday configurations (magnetic field aligned parallel or perpendicular to the quantum well plane, respectively). It is evident that while there is a strong dependence of P_{th} on magnetic field in Faraday configuration, no substantial variations in P_{th} is observed in response to magnetic fields up to $B_\parallel = 1.5$ T.

REFERENCES

1. L. V. Keldysh, A. N. Kozlov, Collective properties of excitons in semiconductors. *Sov. Phys. JETP* **27**, 521–528 (1968).

2. S. A. Moskalenko, D. W. Snoke, *Bose-Einstein Condensation of Excitons and Biexcitons and Coherent Nonlinear Optics with Excitons* (Cambridge Univ. Press, 2010).

3. D. W. Snoke, Spontaneous Bose coherence of excitons and polaritons. *Science* **298**, 1368–1372 (2002).

4. L. V. Butov, A. C. Gossard, D. S. Chemla, Macroscopically ordered state in an exciton system. *Nature* **418**, 751–754 (2002).

5. D. Snoke, S. Denev, Y. Liu, L. Pfeiffer, K. West, Long-range transport in excitonic dark states in coupled quantum wells. *Nature* **418**, 754–757 (2002).

6. S. Yang, A. T. Hammack, M. M. Fogler, L. V. Butov, A. C. Gossard, Coherence length of cold exciton gases in coupled quantum wells. *Phys. Rev. Lett.* **97**, 187402 (2006).

7. A. V. Gorbunov, V. B. Timofeev, Large-scale coherence of the Bose condensate of spatially indirect excitons. *JETP Lett.* **84**, 329–334 (2006).

8. Yu. E. Lozovik, I. L. Kurbakov, G. E. Astrakharchik, J. Boronat, M. Willander, Strong correlation effects in 2D Bose-Einstein condensed dipolar excitons. *Solid State Commun.* **144**, 399–404 (2007).

9. M. Combescot, O. Betbeder-Matibet, R. Combescot, Bose-Einstein condensation in semiconductors: The key role of dark excitons. *Phys. Rev. Lett.* **99**, 176403 (2007).

10. B. Laikhtman, R. Rapaport, Exciton correlations in coupled quantum wells and their luminescence blue shift. *Phys. Rev. B* **80**, 195313 (2009).

11. A. A. High, J. R. Leonard, A. T. Hammack, M. M. Fogler, L. V. Butov, A. V. Kavokin, K. L. Campman, A. C. Gossard, Spontaneous coherence in a cold exciton gas. *Nature* **483**, 584–588 (2012).

12. R. Combescot, M. Combescot, “Gray” BCS condensate of excitons and internal Josephson effect. *Phys. Rev. Lett.* **109**, 026401 (2012).

13. A. A. High, J. R. Leonard, M. Remeika, L. V. Butov, M. Hanson, A. C. Gossard, Condensation of excitons in a trap. *Nano Lett.* **12**, 2605–2609 (2012).

14. A. A. High, A. T. Hammack, J. R. Leonard, S. Yang, L. V. Butov, T. Ostatnický, M. Vladimirova, A. V. Kavokin, T. C. H. Liew, K. L. Campman, A. C. Gossard, Spin currents in a coherent exciton gas. *Phys. Rev. Lett.* **110**, 246403 (2013).

15. Y. Shilo, K. Cohen, B. Laikhtman, K. West, L. Pfeiffer, R. Rapaport, Particle correlations and evidence for dark state condensation in a cold dipolar exciton fluid. *Nat. Commun.* **4**, 2335 (2013).

16. M. Stern, V. Umansky, I. Bar-Joseph, Exciton liquid in coupled quantum wells. *Science* **343**, 55–57 (2014).

17. M. Alloing, M. Beian, M. Lewenstein, D. Fuster, Y. González, L. González, R. Combescot, M. Combescot. F. Evidence for a Bose-Einstein condensate of excitons. *Europhys. Lett.* **107**, 10012 (2014).

18. M. Beian, M. Alloing, E. Cambril, C. G. Carbonel, J. Osmond, A. Lemaître, F. Dubin, Long-lived spin coherence of indirect excitons in GaAs coupled quantum wells. *Europhys. Lett.* **110**, 27001 (2015).

19. K. Cohen, Y. Shilo, K. West, L. Pfeiffer, R. Rapaport, Dark high density dipolar liquid of excitons. *Nano Lett.* **16**, 3726–3731 (2016).

20. M. Combescot, R. Combescot, F. Dubin, Bose-Einstein condensation and indirect excitons: A review. *Rep. Prog. Phys.* **80**, 066501 (2017).

21. R. Anankine, M. Beian, S. Dang, M. Alloing, E. Cambril, K. Merghem, C. Gomez Carbonell, A. Lemaître, F. Dubin, Quantized vortices and four-component superfluidity of semiconductor excitons. *Phys. Rev. Lett.* **118**, 127402 (2017).

22. Y. Mazuz-Harpaz, K. Cohen, M. Leveson, K. West, L. Pfeiffer, M. Khodas, R. Rapaport, Dynamical formation of a strongly correlated dark condensate of dipolar excitons. *Proc. Natl. Acad. Sci. U.S.A.* **116**, 18328–18333 (2019).

23. Z. Wang, D. A. Rhodes, K. Watanabe, T. Taniguchi, J. C. Hone, J. Shan, K. F. Mak, Evidence of high-temperature exciton condensation in two-dimensional atomic double layers. *Nature* **574**, 76–80 (2019).

24. S. Misra, M. Stern, V. Umansky, I. Bar-Joseph, The role of spin-flip collisions in a dark-exciton condensate. *Proc. Natl. Acad. Sci. U.S.A.* **119**, e2203531119 (2022).

25. K. Kowalik-Seid, X. P. Vögele, B. N. Rimpfl, S. Manus, J. P. Kotthaus, D. Schuh, W. Wegscheider, A. W. Holleitner, Long exciton spin relaxation in coupled quantum wells. *Appl. Phys. Lett.* **97**, 011104 (2010).

26. A. Abragam, L. C. Hebel, The principles of nuclear magnetism. *Am. J. Phys.* **29**, 860–861 (1961).

27. D. Paget, G. Lampel, B. Sapoval, V. I. Safarov, Low field electron-nuclear spin coupling in gallium arsenide under optical pumping conditions. *Phys. Rev. B* **15**, 5780–5796 (1977).

28. V. K. Kalevich, K. V. Kavokin, I. A. Merkulov, "Dynamic nuclear polarization and nuclear fields" in *Spin Physics in Semiconductors*, M. I. Dyakonov Ed. (Springer, 2008).

29. D. Gammon, A. L. Efros, T. A. Kennedy, M. Rosen, D. S. Katzer, D. Park, S. W. Brown, V. L. Korenev, I. A. Merkulov, Electron and nuclear spin interactions in the optical spectra of single GaAs quantum dots. *Phys. Rev. Lett.* **86**, 5176–5179 (2001).

30. P. Maletinsky, C. W. Lai, A. Badolato, A. Imamoglu, Nonlinear dynamics of quantum dot nuclear spins. *Phys. Rev. B* **75**, 035409 (2007).

31. A. I. Tartakovskii, T. Wright, A. Russell, V. I. Fal'ko, A. B. Van'kov, J. Skiba-Szymanska, I. Drouzas, R. S. Kolodka, M. S. Skolnick, P. W. Fry, A. Tahraoui, H.-Y. Liu, M. Hopkinson, Nuclear spin switch in semiconductor quantum dots. *Phys. Rev. Lett.* **98**, 026806 (2007).

32. M. N. Makhonin, K. V. Kavokin, P. Senellart, A. Lemaître, A. J. Ramsay, M. S. Skolnick, A. I. Tartakovskii, Fast control of nuclear spin polarization in an optically pumped single quantum dot. *Nat. Mater.* **10**, 844–848 (2011).

33. B. Urbaszek, X. Marie, T. Amand, O. Krebs, P. Voisin, P. Maletinsky, A. Högele, A. Imamoglu, Nuclear spin physics in quantum dots: An optical investigation. *Rev. Mod. Phys.* **85**, 79–133 (2013).

34. G. Sallen, S. Kunz, T. Amand, L. Bouet, T. Kuroda, T. Mano, D. Paget, O. Krebs, X. Marie, K. Sakoda, B. Urbaszek, Nuclear magnetization in gallium arsenide quantum dots at zero magnetic field. *Nat. Commun.* **5**, 3268 (2014).

35. E. A. Chekhovich, A. Ulhaq, E. Zallo, F. Ding, O. G. Schmidt, M. S. Skolnick, Measurement of the spin temperature of optically cooled nuclei and GaAs hyperfine constants in GaAs/AlGaAs quantum dots. *Nat. Mater.* **16**, 982–986 (2017).

36. The excitation energy of laser is well below the bandgap of top and bottom contact layers and the barriers between the CQW and contact layers. Hence, the photodepletion mechanism giving rise to the ring formation of (*4*) and (*5*) is suppressed.

37. The saturation of ΔE with power is due to the appearance of the Z line (*16*). This high-power regime is not discussed in this work.

38. M. Dobers, K. Klitzing, J. Schneider, G. Weimann, K. Ploog, Electrical detection of nuclear magnetic resonance in GaAs−$Al_xGa_{1-x}As$ heterostructures. *Phys. Rev. Lett.* **61**, 1650–1653 (1988).

39. G. Petersen, E. A. Hoffmann, D. Schuh, W. Wegscheider, G. Giedke, S. Ludwig, Large nuclear spin polarization in gate-defined quantum dots using a single-domain nanomagnet. *Phys. Rev. Lett.* **110**, 177602 (2013).

40. M. Kotur, F. Saeed, R. W. Mocek, V. L. Korenev, I. A. Akimov, A. S. Bhatti, D. R. Yakovlev, D. Suter, M. Bayer, Single-beam resonant spin amplification of electrons interacting with nuclei in a GaAs/(Al,Ga)As quantum well. *Phys. Rev. B* **98**, 205304 (2018).

41. M. Tavis, F. W. Cummings, Exact solution for an *N*-molecule—Radiation-field Hamiltonian. *Phys. Rev.* **170**, 379–384 (1968).

42. Y. Kubo, C. Grezes, A. Dewes, T. Umeda, J. Isoya, H. Sumiya, N. Morishita, H. Abe, S. Onoda, T. Ohshima, V. Jacques, A. Dréau, J.-F. Roch, I. Diniz, A. Auffeves, D. Vion, D. Esteve, P. Bertet, Hybrid quantum circuit with a superconducting qubit coupled to a spin ensemble. *Phys. Rev. Lett.* **107**, 220501 (2011).

43. C. Grèzes, "Towards a spin ensemble quantum memory for superconducting qubits," thesis, University of Paris VI, France (2016).

44. S. Misra, M. Stern, A. Joshua, V. Umansky, I. Bar-Joseph, Experimental study of the exciton gas-liquid transition in coupled quantum wells. *Phys. Rev. Lett.* **120**, 047402 (2018).
